# Can the Hydroxyapatite-Coated Skin-Penetrating Abutment for Bone Conduction Hearing Implants Integrate with the Surrounding Skin?

**DOI:** 10.3389/fsurg.2015.00045

**Published:** 2015-09-14

**Authors:** Marc van Hoof, Stina Wigren, Hans Duimel, Paul H. M. Savelkoul, Mark Flynn, Robert Jan Stokroos

**Affiliations:** ^1^Department of Otorhinolaryngology and Head and Neck Surgery, Maastricht University Medical Center, Maastricht, Netherlands; ^2^Cochlear Bone Anchored Solutions AB, Mölnlycke, Sweden; ^3^Institute of Nanoscopy, Maastricht University Medical Center, Maastricht, Netherlands; ^4^Department of Medical Microbiology, NUTRIM School of Nutrition and Translational Research in Metabolism, Maastricht University Medical Center, Maastricht, Netherlands; ^5^Department of Medical Microbiology and Infection Control, VU University Medical Center, Amsterdam, Netherlands

**Keywords:** skin integration, bone conduction hearing implant, hydroxyapatite, adverse skin reactions, SEM, histology, Baha

## Abstract

**Introduction:**

Percutaneous implants, such as bone conduction hearing implants, suffer from complications that include inflammation of the surrounding skin. A sealed skin–abutment interface can prevent the ingress of bacteria, which should reduce the occurrence of peri-abutment dermatitis. It was hypothesized that a hydroxyapatite (HA)-coated abutment in conjunction with soft tissue preservation surgery should enable integration with the adjacent skin. Previous research has confirmed that integration is never achieved with as-machined titanium abutments. Here, we investigate, *in vivo*, if skin integration is achievable in patients using a HA-coated abutment.

**Materials and methods:**

One titanium abutment (control) and one HA-coated abutment (case) together with the surrounding skin were surgically retrieved from two patients who had a medical indication for this procedure. Histological sections of the skin were investigated using light microscopy. The abutment was qualitatively analyzed using scanning electron microscopy.

**Results:**

The titanium abutment only had a partial and thin layer of attached amorphous biological material. The HA-coated abutment was almost fully covered by a pronounced thick layer of organized skin, composed of different interconnected structural layers.

**Conclusion:**

Proof-of-principle evidence that the HA-coated abutment can achieve integration with the surrounding skin was presented for the first time.

## Introduction

For many decades, research has focused on enhancing clinical outcomes with percutaneous (skin-penetrating) implants, which have a broad application in medicine (1–3). Persistent or recurring inflammation and/or infection of the soft tissues around the implant lead to significant morbidity. These complications are also an important factor in the associated costs of these devices (4). The mechanisms of the inflammatory reaction around a skin-penetrating device are not completely understood, but are supposed to include infections by bacterial and fungal pathogens (5, 6), a foreign body reaction (FBR) (7, 8), and shear stresses from surrounding soft tissues (9, 10). The current leading hypothesis and consensus is that “sealing” the skin–implant interface should lead to better clinical outcomes. This could possibly be achieved by impeding the epidermis from migrating downwards alongside the implant (11). A healthy dermal collagenous matrix and epithelial cells attached to the implant could provide a natural border inhibiting this migration (2, 12). The tight connection between the viable host tissues and the implant is believed to be the key in preventing the formation of a moist pocket around the implant and preventing pathogens from colonizing the implant surface.

It is believed that skin integration is a race between the host’s skin and bacterial growth on the implant surface (13, 14). Whichever comes first or is the most pronounced could determine the clinical outcome. *In vitro*, as a proxy for the ability of human skin tissues to integrate to implants, the ability of keratinocytes to adhere and proliferate on different implant materials have been investigated (15). The translation of these findings, which are often contradictive (16), to clinical applications have not been forthcoming. A promising biomaterial, hydroxyapatite (HA), has been proposed to serve as a biocompatible coating over the implant and to integrate with the skin (17). After evidence of achieving skin integration in animals (12), Kang et al. claim to have developed HA-coated flanged implants that were able to integrate to the human skin in both limb (18) and extra-oral craniofacial prostheses (19). Although featuring a small number of subjects and relying on subjective surgical outcome measures, these studies showed the potential of HA for cutaneous integration.

The bone conduction hearing implant system, previously referred to as a Bone-Anchored Hearing Aid (20), is under investigation here. It is a system that is worn behind the ear comprising an osseointegrated implant with a percutaneous abutment attached. An external sound processor converts sound to vibrations that elicit the perception of sound in the cochlea via bone conduction. The sound processor can be coupled to the abutment by patients as they please. Today, more than 100,000 patients worldwide use this system in order to improve their hearing. The system suffers from the same problems as other percutaneous implants with peri-abutment dermatitis occurring in up to 38% of patients (21). Nowadays, as an alternative to a percutaneous abutment, several new transcutaneous systems exist where the skin is not permanently breached (22). These solutions are promising in their complication rates and cosmetic aspects but because of different amplification needs for distinct patient groups they do not remove the necessity of a percutaneous system.

Over the years, research was primarily focused on enhancing the surgical technique for placement of the percutaneous bone conduction hearing implant (23). These techniques all included soft tissue reduction to reduce skin motion and limit the formation of deep epidermal pockets, which was believed to be essential to maintaining acceptable complication rates. As it turns out, solely adapting the surgical procedure did not bring the incidence of inflammation, as classified by the Holgers grade (24), down to the rate of its physiological counterparts, such as teeth (25). Previously, it has been concluded that soft tissue integration is not established with all-titanium bone conduction hearing implants (6). The surface of removed as-machined titanium abutments was mostly characterized by the absence of attaching cells. An amorphous layer, supposedly composed of proteins, bacteria, fungi, leukocytes and shed, keratinized epithelial cells, was sometimes observed (6). This amorphous material has also been associated with biofilm (26).

Although a strict definition of soft tissue integration is lacking, the current consensus includes physical locking by a viable and vascularized layer of tissue tightly connecting the implant surface to the host to prevent microbial colonization (13, 27, 28).

**Figure 1:**
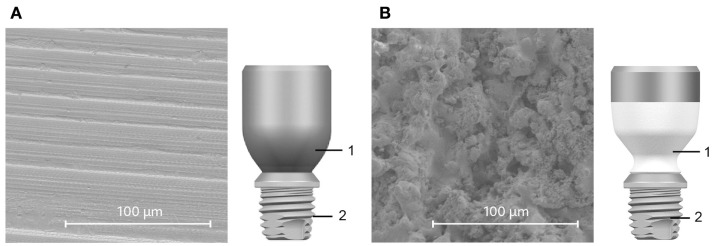
**Comparison of abutments (1)**. The all-titanium Cochlear Baha BA300 Abutment **(A)** and HA-coated BA400 abutment **(B)** are shown alongside SEM images of their respective surfaces. The partial HA coating is white in the illustration **(B)**. Both abutments connect to the same implant fixture (2).

Recently, one of the manufacturers of bone conduction hearing implant systems introduced an HA-coated titanium abutment (Figure 1). It is designed for soft tissue integration and histological evidence of an intimate tissue-to-abutment contact was previously shown in an animal study (29). In contrast to all of its titanium predecessors, the HA-coated abutment was able to form dermal attachments and reduce epidermal downgrowth. Both can be seen as signs of skin integration (29).

To our knowledge, this is the first soft tissue integration project that is designed to translate and substantiate findings from basic research to its clinical application. Within this project, proof-of-principle and exploratory clinical investigations evaluating the skin integration at structural and ultrastructural levels are complemented by a large-scale randomized controlled trial focusing on clinical and health-economic parameters in which over 100 patients have already been operated on (30). The present proof-of-principle study aims to determine if the percutaneous HA-coated abutment can achieve skin integration in patients.

## Materials and Methods

### Ethics

The procedures in this investigation were in accordance with legislation (the Medical Research Involving Human Subjects Act) and ethical standards on human experimentation in the Netherlands. No approval was sought from an ethics committee beforehand as it is not required when using materials derived from anonymized patients that can be considered waste products from surgery. Only patients who primarily had a medical indication for the surgical removal of the abutment, including the surrounding skin, were invited to participate. Verbal informed consent was gained prior to the removal for the specific aim of informing the patients that their data and materials would be collected during surgery. The informed consent was noted in the electronic patient dossier. The materials and patient records were subsequently coded and anonymized. The individuals whose samples are presented in this manuscript have provided written informed consent to publish these case details.

### Implants

Two types of abutments were evaluated in this study. The 6-mm-long titanium abutment (Cochlear™ Baha^®^ BA300 Abutment, Cochlear Bone Anchored Solutions AB, Mölnlycke, Sweden) is manufactured from medical grade titanium (Figure 1A) and features an as-machined surface finish. The 10-mm-long HA-coated titanium abutment (Cochlear Baha BA400 Abutment, Cochlear Bone Anchored Solutions AB, Mölnlycke, Sweden) is also manufactured from medical grade titanium (Figure 1B) but features a plasma-sprayed HA coating. The HA coating covers 7 mm of the total abutment length starting from the base. The upper titanium part of this abutment, which is intended to protrude above skin level, is not coated. Both abutments are supplied mounted on a Cochlear Baha BI300 Implant fixture.

### Scanning electron microscopy

The retrieved abutments were directly fixed after removal in a 3% glutaraldehyde 0.1 M phosphate buffer (PB). These were rinsed in PB and subsequently dehydrated in ascending concentrations of alcohol (70 and 90% for 15 min and twice in 100% alcohol for 30 min). The samples were critical-point dried and fixed to a mount with carbon paint and metalized with gold. The presence of attached tissues and cells was visually inspected using a Philips XL30^®^ scanning electron microscope (Philips, Eindhoven, the Netherlands) on both abutments using high magnifications.

### Light microscopy

For histology, tissue specimens were fixed in 4% paraformaldehyde 0.1M PB at room temperature. The samples were washed with 0.09 M monopotassium phosphate +7.5% sucrose to avoid chemical reactions between glutaraldehyde and osmium. Afterwards, they were post fixed with 1% osmium tetraoxide in a veronal buffer +1.5% ferrocyanide pH 7.4 at 4°C for 1 h. They were rinsed in a veronal buffer +7% sucrose at 4°C for 5 min. The specimens were subsequently dehydrated in ascending concentrations of alcohol (70% and 90% for 15 min and twice in 100% alcohol for 30 min). The specimens were placed twice in propylene oxide (PO) for 30 min. After this step, they were kept in a new resin embedding (Epon mix with PO 1:1) overnight. On the next day, the samples were embedded in an embedding capsule and polymerized with fresh Epon for 72 h at 50°C. After fixation, the samples were cut into 1-μm slides and stained with toluidine blue. Examinations were performed using an Olympus BX51 microscope (Olympus Europe, Hamburg, Germany).

## Results

### Patient reports

Patient 1 (Table 1) was a 25-year-old female implanted with the titanium abutment 2 years previously. The abutment was placed using the recommended surgical procedure at the time, which was a linear incision with soft tissue thinning (31). Her history of disease included constitutional eczema and otitis externa. She had recurring and persistent episodes of peri-abutment dermatitis, which were clinically unrelated to the constitutional eczema. The inflammation was unresponsive to topical and systemic antibiotics (classified as a Holgers grade 4). This eventually led to the decision to remove the abutment. At the time of removal, the inflammation was moderate (classified as a Holgers grade 2 reaction) for which she was being treated with a topical ointment (Nasumel ^®^, Bfactory Health Products B.V. Rhenen, The Netherlands). At that point in time, the soft tissue thickness was approximately 4 mm. The abutment was removed under local anesthesia by unscrewing its internal screw that mounts it to the osseointegrated implant fixture. The abutment was not attached to the surrounding soft tissue. A thin layer of surrounding tissue was excised using a scalpel.

**Table 1:** **Demographics and clinical outcomes of two Baha users**.

	Patient 1	Patient 2
Age (years)	25	68
Sex	Female	Male
Relevant underlying conditions and medication usage	Constitutional eczema, otitis externa.	–
Type of implant	Titanium abutment (BA300 Abutment)	HA-coated abutment (BA400 Abutment)
Abutment in place (years)	2	1
Surgical placement technique	Retro-auricular linear incision with soft tissue thinning.	Retro-auricular linear incision without soft tissue thinning.
Reason for removal	Severe, recurring, and persistent peri-abutment dermatitis (Holgers grade 4).	Received a Cochlear Implant on the side of the Baha.
Complications related to the implant	Severe, recurring, and persistent peri-abutment dermatitis (Holgers grade 4). On removal, only redness and moistness were present (Holgers grade 2).	Several episodes of redness, moistness, and granulation formation around the Baha (Holgers grades 1–3). At the time of removal, the skin adjacent to the Baha was only mildly red (Holgers grade 1).

Patient 2 (Table 1) was a 68-year-old male with no relevant history of disease who had been implanted with an HA-coated abutment using a linear incision without soft tissue thinning (32) one year prior to removal. The patient developed minor to moderate peri-abutment dermatitis several times during the first few months post-operatively, which were successfully treated with systemic and topical antibiotics. The patient was found eligible for a cochlear implant (CI) on the side of the abutment that indicated surgical removal. Upon removal, the patient had a minor redness (classified as a Holgers grade 1) around the abutment with a caudal pocket, which resulted from a prior episode of peri-abutment dermatitis. The soft tissue thickness was approximately 4 mm. The abutment was removed under general anesthesia by unscrewing its internal screw that mounts it to the osseointegrated implant fixture. A thin layer of surrounding tissue was excised using a scalpel after removal of the abutment. The surgical procedure was combined with the implantation of the CI.

### Scanning electron microscopy

#### The Titanium Abutment

**Figure 2:**
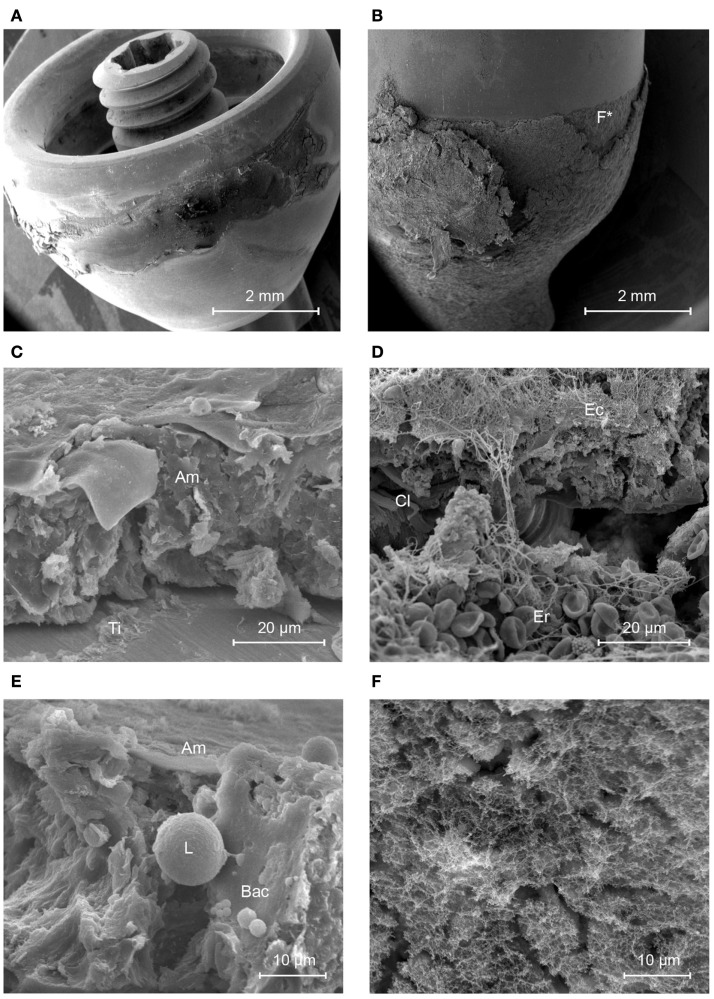
**Scanning electron micrographs of the titanium and HA-coated abutment**. **(A)** Titanium abutment. **(B)** HA-coated abutment. **(C)** Structured soft tissue is not present on the titanium abutment (view from the top of the abutment). **(D)** The sharply delineated area of HA is covered by multiple layers of soft tissues containing collagen fibers (Cl), Erythrocytes (Er), and connected epithelium (Ec) as opposed to its titanium surface (Ti). **(E)** The amorphous layer (Am) that is present on the titanium surface shows a group of planktonic coccoid bacteria (Bac) in presence of a leukocyte (L). **(F)** This image is a higher magnification of the area with disrupted epithelial coverage in **(B)** (F*) and shows a layer containing a matrix of proteins.

The bare BA300 titanium abutment surface was mostly covered by a thin, unstructured amorphous material thought to be composed of proteins, lipids, sebum, keratin, compacted cornified keratinocytes, leukocytes, and components of biofilm such as extracellular polymeric substance (EPS) (Figure 2). Planktonic bacteria were also seen (Figure 2E).

#### The HA-Coated Abutment

**Figure 3:**
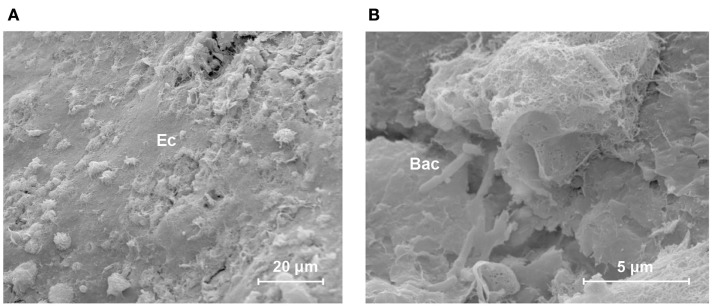
**Host defense in response to bacteria in the skin–implant interface of the HA-coated abutment**. This scanning electron micrograph shows an overview of the different connected, non-shedding epithelial cells (Ec) on the outer surface of the HA coating [**(A)** lateral view)]. Immune cells and bacteria are seen. A close-up view [**(B)** scale bar = 5 μm] shows ongoing phagocytosis in the neighborhood of planktonic rod-shaped bacteria (Bac).

The HA-coated abutment (Figure 2B) appeared to be completely covered by biological material, solely on the parts of the surface that were coated with HA. The titanium part of the HA-coated abutment was free of attached tissues. The HA coating was covered by organized, viable layers of keratinocytes and a layer of collagen fibers (Cl) (Figure 2D). Only a small amount of epithelial cells, at the upper regions of the HA coating, showed signs of cornification and detachment from their surroundings. There was no presence of accumulation of non-viable corneocytes or cellular debris. Planktonic bacteria undergoing phagocytosis were seen, which is evidence of an active immune response (Figure 3). There were no signs of generalized biofilm formation. There also seemed to be a protein matrix attached directly to the HA or to a layer of cells in intimate contact with the HA surface (Figure 2F).

### Histology

#### The Titanium Abutment

**Figure 4:**
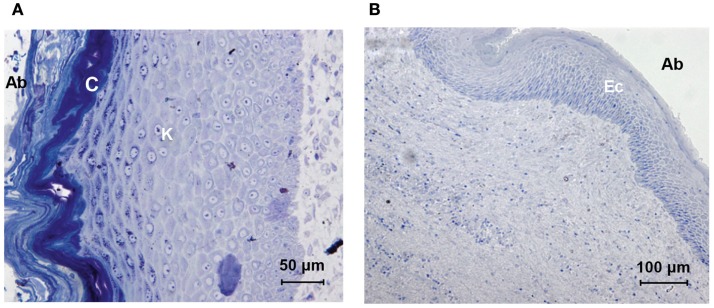
**Histologic comparison of skin specimens around the titanium and HA-coated abutment**. The skin directly surrounding the titanium abutment [**(A)** oblique orientation, abutment-facing surface to the left (Ab)] shows extensive stratified keratinization (K) and cornification (C). The skin directly surrounding the HA-coated abutment [**(B)** longitudinal orientation, abutment-facing surface to the right, (Ab)] shows viable stratified layers of epithelial cells (Ec); no keratinization or shedding is present.

The section of the skin around the titanium abutment shows epidermal stratification (Figure 4A). The stratum corneum, stratum lucidum, stratum granulosum, stratum spinosum, and stratum basale with the subsequent transition to the dermis can be seen. Keratinization increases after the stratum granulosum. The stratum corneum, which is the outer abutment-facing layer, has a strong presence of cornification and squamation. The total thickness of all the epidermal layers was approximately 300 μm. There were no signs of extensive granulation. Dermal papilla or hair follicles were absent.

#### The HA-Coated Abutment

The abutment-facing epidermal layers were stratified and non-keratinized (Figure 4B). The epidermis, facing the abutment lacked rete pegs, had a viable flattened outer layer containing nuclei and there were no signs of cornification or squamation. The stratum spinosum and stratum basale could be identified. The total thickness of all the epidermal layers was approximately 120 μm. Dermal papilla and hair follicles were absent.

## Discussion

### Comparison of results

In summary, the results for these two patients are distinct. The scanning electron microscopy (SEM) analysis showed viable tissue in intimate contact with the HA coating, indicating effective skin integration and including signs of an effective immune system. Such findings were lacking on the titanium abutment or on the top titanium portion of the HA-coated abutment. The histological analysis showed that the abutment-facing cell layers only had nuclei in the case of the HA-coated abutment. There was no cornification or keratinization present. This can be considered a prerequisite for the establishment of a viable direct soft tissue-to-abutment contact.

### Skin integration, microbiota, and the immune response

A long-term percutaneous implant, starting from its surgical placement, endures continuous colonization attempts by microorganisms. The resulting bacterial selection pressure probably leads to a distinct, highly specialized, multi-species microbiota possibly in the form of a biofilm on (26) and around the abutment. This could provide a partial explanation for the recurrent or persistent inflammations often encountered in clinical practice. Therefore, substantial effort is spent on designing implant materials that resist bacterial colonization, either by bacterial resistant coatings (14) or by ensuring that there is a skin seal (15). Holgers et al. (6) previously showed that seven out of nine titanium abutments had no macroscopically visible materials and no cells attaching to the abutment surface. Some titanium abutments showed evidence of amorphous materials, bacteria and fungi, leukocytes and shed keratinized cells, similar to the results presented here. They concluded that there was no viable attachment in any of the titanium abutments studied. Mlynski et al. (33) also concluded that soft tissue does not attach tightly to the titanium bone conduction hearing implant abutment. Their histological results showed no signs of skin integration. The skin surrounding the titanium abutments showed normal keratinization or even hyperkeratosis, similar to our results. Although the function of the skin’s stratum corneum is to expel bacteria (34), the presence of a percutaneous abutment could inhibit the clearance of shed corneocytes into the peri-abutment pocket that results from the non-adherence of the tissue to the abutment. This would allow for accumulation of non-viable materials in which bacteria seem to prosper and could explain the observation of amorphous structures on top of the titanium abutment surface in the present investigation. Similar observations have previously been made around abutments for bone conduction hearing implants and are described as biofilm by Monksfield et al. (26).

The role of the immune system in skin integration has received less attention. The immune response should not be suppressed, as this reduces its capacity to combat infections (13, 14). To maintain a healthy implant site, the current consensus is that vascularized soft tissue integration not only provides a physical barrier to bacteria (28) but also enables the host’s immune defense or systemic antibiotics to reach areas sensitive to bacterial invasion. It has been shown *in vivo* that smooth surfaces have a higher risk of infection than rough, porous surfaces that display soft tissue ingrowth (27). At the same time, it is known that bacteria prefer a rough surface (35). The discrepancy between these two observations could possibly be explained by the role of the host’s immune system. When the host’s immune system is not inhibited or compromised by the avascular physical barrier of strong cornification that can form around titanium abutments, it could possibly be more capable of preventing and healing infections by means of enhanced access.

Moreover, a barrier without cornification would mimic the physiological junctional epithelium around teeth or the one that develops around dental implants (6, 36, 37). Junctional epithelium is a highly specialized cell layer whose function is to bridge the soft tissues to teeth while acting as a barrier which is permeable for an immune response to prevent bacteria in dental plaques from invading the host further (36). Several features of junctional epithelium correspond to observations made when analyzing the soft tissues around the HA-coated abutment in this investigation. Examples include the prominent immune recruitment in response to bacteria and viable peri-abutment epithelia without keratinization and cornification. In this context, it is interesting to note that junctional epithelium can form *de novo* from gingival oral keratinocytes by phenotypic change (38). Phenotypic change may be hypothesized to also play a role in the skin integration seen with HA-coated abutments.

### Shear stresses and physical locking

The tolerance of the integrated skin–implant interface to push and pull forces could be important in the advent of inflammation (39). Holt et al. (39) suggested that three different regions develop around the implant to cope with these forces. The *interface region* serves as a first attachment. The *transition region* bridges the interface region and the *stress-absorbance region*. The latter functions as a buffer for “push and pull” forces between the rigid fixated implant and the surrounding tissues. These regions could also be partially identified here in the tissue surrounding and covering the HA-coated abutment. The interface region could be present in the form of a basal lamina attached to the HA of which the observed protein matrix would be a part. An alternative explanation would be that fibroblasts created an extracellular matrix for epidermal cells to connect to (11). The ability of epithelial cells to attach to titanium implants using hemidesmosomes or a basal lamina has been shown before (40–42). The attachment is expected to have a strong relationship to wound healing as discussed in the next paragraph. The transition region and stress-absorbance region are less distinctly characterized in the results presented here. Collagen fibers can have stress-absorbent properties and were also observed in the layer of tissue on top of the HA-coated abutment. But their function and orientation is not yet clear around implants (43, 44).

Both abutments easily detached from the surrounding tissue during the removal procedure. In line with the other results, this was to be expected for the titanium abutment. Clinicians have been manually replacing titanium abutments for decades. By contrast, a layer of tissue still remained directly attached to the HA-coated part of the HA-coated abutment. This suggests that the linkage between the HA-coated abutment and the directly attaching tissues is stronger than the connection between the latter and the surrounding skin.

### Wound healing and skin integration

The stage is set for skin integration when a wound is surgically created. The host’s healing response quickly commences with blood clotting on the abutment surface. Over time, granulation tissue is formed around the abutment and keratinocytes can be expected to migrate for re-epithelialization. Upon reaching the abutment, components from both the epidermis and dermis can be responsible for the actual attachment as previously discussed. Residing in the granulation tissue, fibroblasts can attach to the abutment surface and create an extracellular matrix (42). From the epidermis, migrating keratinocytes could possibly use hemidesmosome complexes to attach to the abutment directly or by connecting to an extracellular matrix surrounding the abutment that has been previously created. The attached tissues on the HA-coated abutment here could support either theory. On the host side of the interface, keratinocytes can connect to each other using desmosomes to close the breach of skin integrity that was the end-goal of wound healing and skin integration.

### Future studies

The present proof-of-principle study allows for assessing the ultrastructural composition and degree of attachment. It does not allow for a conclusive identification of the different cells and layers and how these are interconnected. Moreover, it is not unlikely that the attachment differs within and between patients in relation to (traumatic) skin shear stresses, time, complications, and patient characteristics. Future studies are needed to investigate if the observations and hypotheses presented in this paper can be further corroborated by immunological, microbiological (45), and molecular cell biology research. This could result in a clearer definition of what skin integration is or should be.

Whether the observed skin integration of the HA-coated abutment also results in an enhanced clinical outcome in terms of a reduction in inflammatory reactions has not been investigated here. This is currently being investigated in a randomized controlled trial that compares the clinical outcomes in patients with an HA-coated abutment placed using tissue preservation surgery and patients with a titanium abutment placed using traditional soft tissue reduction techniques (30).

### Limitations

The aim of the study was not to confirm that current titanium abutments are unable to achieve soft tissue integration since this has been shown before (6, 7). Indeed, this investigation solely focused on providing a proof-of-principle that the HA-coated abutment can achieve soft tissue integration in humans. The patients described here have possible confounding factors, such as their history, age, the different surgical procedure, and clinical course. Therefore, ideally, the results should be confirmed in a statistically powered sample size where the two different abutments are placed in two randomly selected groups using an equal surgical procedure; however, conducting such research is difficult due to practical and ethical limitations.

## Conclusion

Evidence that the percutaneous HA-coated abutment for bone conduction hearing implants can achieve integration with the surrounding skin in human subjects was presented for the first time. An ongoing prospective comparative clinical trial should establish if it also leads to a reduced incidence or severity of inflammatory skin reactions. These “proof-of-principle” results may also be of interest to other long-term percutaneous implants in medicine as well.

## Conflict of Interest Statement

We acknowledge a research grant from Cochlear Bone Anchored Solutions AB, which supported this investigator-initiated study. Stina Wigren and Mark Flynn are paid employees of Cochlear Bone Anchored Solutions AB, Mölnlycke, Sweden.
